# Assessment of a multimedia-based prospective method to support public deliberations on health technology design: participant survey findings and qualitative insights

**DOI:** 10.1186/s12913-016-1870-z

**Published:** 2016-10-26

**Authors:** P. Lehoux, J. Jimenez-Pernett, F. A. Miller, B. Williams-Jones

**Affiliations:** 1 Department of Health Management, Evaluation and Policy, University of Montreal, Institute of Public Health Research of University of Montreal (IRSPUM), University of Montreal Research Chair on Responsible Innovation in Health, Branch Centre-ville, P.O. Box 6128, Montreal, QC H3C 3 J7 Canada; 2 Department of Health Management, Evaluation and Policy, University of Montreal, IRSPUM, Montreal, Canada; 3 Institute of Health Policy, Management and Evaluation, University of Toronto, Toronto, Canada; 4 Department of Social and Preventive Medicine, University of Montreal, IRSPUM, Montreal, Canada

**Keywords:** Public involvement, Knowledge transfer and exchange, Evaluation, Video-based deliberations, Online forum, Health innovation, Prospective analyses

## Abstract

**Background:**

Using a combination of videos and online short stories, we conducted four face-to-face deliberative workshops in Montreal (Quebec, Canada) with members of the public who later joined additional participants in an online forum to discuss the social and ethical implications of prospective technologies. This paper presents the participants’ appraisal of our intervention and provides novel qualitative insights into the use of videos and online tools in public deliberations.

**Methods:**

We applied a mixed-method study design. A self-administered survey contained open- and close-ended items using a 5-level Likert-like scale. Absolute frequencies and proportions for the close-ended items were compiled. Qualitative data included field notes, the transcripts of the workshops and the participants’ contributions to the online forum. The qualitative data were used to flesh out the survey data describing the participants’ appraisal of: 1) the multimedia components of our intervention; 2) its deliberative face-to-face and online processes; and 3) its perceived effects.

**Results:**

Thirty-eight participants contributed to the workshops and 57 to the online forum. A total of 46 participants filled-in the survey, for a response rate of 73 % (46/63). The videos helped 96 % of the participants to understand the fictional technologies and the online scenarios helped 98 % to reflect about the issues raised. Up to 81 % considered the arguments of the other participants to be well thought-out. Nearly all participants felt comfortable sharing their ideas in both the face-to-face (89 %) and online environments (93 %), but 88 % preferred the face-to-face workshop. As a result of the intervention, 85 % reflected more about the pros and cons of technology and 94 % learned more about the way technologies may transform society.

**Conclusions:**

This study confirms the methodological feasibility of a deliberative intervention whose originality lies in its use of videos and online scenarios. To increase deliberative depth and foster a strong engagement by all participants, face-to-face and online components need to be well integrated. Our findings suggest that online tools should be designed by considering, one the one hand, the participants’ self-perceived ability to share written comments and, on the other hand, the ease with which other participants can respond to such contributions.

## Background

As more complex forms of health intervention continually emerge, scholars have increasingly voiced arguments in favor of including the public in discussing the putative benefits and risks of innovative technologies [1–7]. While public engagement often occurs late in the design process, i.e., when technologies are actually entering healthcare systems, it may also happen earlier [8]. To this end, a Dutch team [9–11] developed a prospective method to support reflective deliberations about social and technological change that may take shape in the future.

Inspired by this approach, our team designed a study that put forward an “audiovisual-elicitation-based” [12] data collection strategy whose overall goal was to examine the ways in which public deliberations of prospective scenarios can enable a critical examination of the social and ethical issues underlying the design of new health technologies. We conducted four face-to-face deliberative workshops with members of the public who later joined additional participants through an asynchronous online forum. Participants, who resided near Montreal (the largest city in the French speaking Canadian province of Quebec), were invited to discuss scenarios unfolding in the near future of 2030-40, in three areas: enhancement technologies in teenagers, preventive interventions for genetically “at risk” adults and ageing in a high-tech world.

The full protocol of this three-year study can be found here [13]; Table 1 indicates its substantive and methodological objectives. Since the originality of the deliberative intervention at the heart of our study lay in the use of a combination of multimedia material (i.e., videos and online short stories), the aim of the current paper is primarily methodological. More specifically, to provide insights into the use of videos and online tools for fostering critical and reflective deliberations around issues arising with complex health innovations, the evaluation presented in this paper relies on a mixed-method study design. Qualitative data are used to illustrate and flesh out the survey data describing the participants’ appraisal of: 1) the multimedia components of our intervention; 2) its deliberative face-to-face and online processes; and 3) its perceived effects.

**Table 1:** The rationale and objectives of the three-year study

The three thematic areas
• Our broader study aims to generate substantive knowledge about the social and ethical issues raised by new health technology and methodological knowledge on the use of multimedia-based tools in public deliberation.
• We chose to structure the study around thematic areas, not specific diseases or technologies, in order to address a large spectrum of usability and ethical issues, while considering the needs and preferences of different social groups across the life course: 1) the use of enhancement technologies in teenagers; 2) preventive interventions for “at risk” adults; and 3) ageing in a high-tech world.
Substantive objective 1
• To analyze the ways in which members of the public, in face-to-face and online multimedia-based deliberative environments, reason and deliberate about the desirability of technical and social changes that may affect the three thematic areas within a 25-year timeframe;
• *Rationale*: The thematic areas offer enough diversity to explore the subtleties, prejudices and nuances by which participants may ponder normative issues in different contexts, for different human beings (i.e., increasing teenagers’ performance vs. offsetting elderly people’s frailty).
Substantive objective 2
• To identify the usability and ethical issues raised by various design assumptions (e.g., intended use, complexity, impact on autonomy) and features (e.g., accuracy, immediacy, invasiveness, costliness) in these three thematic areas;
• *Rationale*: The common thread behind the thematic areas is that they all address the technological redefinition of “normal” cognitive and physical states and processes, and the growing emphasis on the ability to exercise agency over one’s body.
Methodological objective
• To assess the extent to which the sociotechnical scenario method fosters critical, reflective and creative reasoning and deliberations regarding the design of health innovations.
• *Rationale*: Prospective scenarios enable non-experts to envision and relate to potential futures, possibly fostering through an immersion in fiction their creativity and reflexivity about the practical and moral implications of technological and social developments in health care.

Rigorous, small-scale studies like the one we present are important for scholars and practitioners of public involvement and Knowledge Transfer & Exchange (KTE) who call for structured evaluation approaches in these closely interconnected domains [14–21]. Public involvement and KTE initiatives often share the aim of enabling participants to develop new knowledge and competencies [14] and scholars in both domains have begun examining how online tools may support meaningful and informed deliberations [22–27]. Hence, by providing a theoretically-grounded assessment of a multimedia-based deliberative intervention that sequentially integrated both face-to-face and online components, we aim to contribute to the growing body of methodological literature that examines how public deliberative processes and tools can be improved [19–21].

The paper is comprised of four parts. We first clarify how we structured the evaluation of our deliberative intervention, making explicit its underlying “theory” and clarifying how its components and processes were expected to affect participants [20]. Second, we describe the quantitative and qualitative data that we collected and analyzed. Third, we examine the extent to which the videos and online scenarios helped participants understand the context in which three fictional technologies would be used as well as the challenges they posed, and the extent to which the face-to-face and online deliberative environments enabled them to engage in critical and reflective deliberations. Fourth, we discuss how this paper advances current knowledge of how to use various tools in public deliberative processes. Specifically, our findings confirm the methodological feasibility of a deliberative intervention whose originality lies in its use of multimedia-based tools and helps to understand why face-to-face and online environments need to be combined appropriately in order to increase deliberative depth.

### Assessment of tailor-made public involvement initiatives

To fulfill their specific goals and reach their intended audiences, public involvement initiatives usually rely on a combination of strategies and, as a result, often possess a unique set of characteristics [18]. As such, the assessment of these tailor-made initiatives less often relies on standardized instruments [21], and randomized control studies are exceedingly rare [14]. To capture the key characteristics of a given public involvement initiative, Popay, Collins and the Public Involvement Impact Assessment Framework (PiiAF) Study Group recommend using an evaluation framework that makes the “intervention theory” explicit [20]. This theory entails a description of the ways in which a particular approach to involving the public will lead to the expected effects. It is around this intervention theory that one may identify what data to collect and how, in order to document the intervention’s impact [20: 9]. This recommendation is in line with a research gap identified by Abelson and colleagues:

Much of the empirical public engagement evaluation work in the health field continues to be carried out in the absence of any guiding frameworks that define the theoretical basis for the public engagement process or the relationships among the public engagement mechanism and process or outcome variables of interest [14: 10].

For these authors, “building a strong theoretical foundation requires equal attention” to: the definition of the goals and of the context in which the public involvement intervention unfolds; the unpacking of the components supporting each of the goals in order to evaluate the deliberative process; and the clarification of the outcomes of interest, which may include organizational, decision-making, policy and/or participant outcomes [14: 6]. We thus describe below our intervention and its underlying theory.

### The intervention theory underlying our multimedia-based deliberative intervention

#### Goals and context of our broader study

**Fig. 1:**
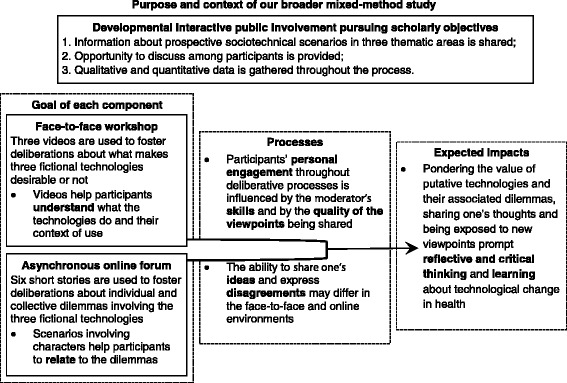
A schematic illustration of our multimedia-based intervention theory

Public involvement may pursue different goals, which can be categorized as either *democratic* when the initiative is “intended to meet transparency, accountability, trust and confidence goals,” *instrumental* when the initiative is “designed to improve the quality of decision-making,” or *developmental* when the initiative is intended “to improve knowledge and capacity of the participants” [14: 19]. As Fig. 1 indicates, our intervention is characterized as developmental; it was designed to enable non-experts to deliberate about complex health innovation issues. To do so, our intervention incorporated three key elements of *interactive* public engagement approaches: 1) information was shared with participants about the issues under discussion; 2) the format allowed interactive discussion among participants; and 3) both individual and collective input were gathered through an explicit, structured process [14]. There were no sponsors, policy-makers or practitioners to whom the results of the deliberations were directed. Participants were invited to provide their input within a research context. Our recruitment tools conveyed to potential participants the full rationale of our study: there are very few tools to examine prospectively how the public define and appraise the desirability of health innovations. This is the gap our broader study intends to bridge and the basis upon which participants agreed to participate.

#### Components

Our intervention relied on three video clips that were discussed in four face-to-face deliberative workshops with members of the public, and six dilemmas that were discussed through an asynchronous online forum with additional participants. This multimedia material was structured to address somewhat audacious, yet empirically plausible sociotechnical changes in three thematic areas. Table 2 provides a summary of the technologies we “invented” for each area, relying on the method^1^ elaborated by Boenink and colleagues for whom prospective scenarios are “historically informed speculations” describing possible futures [8: 6]. The decision to use multimedia material was anchored in the KTE literature and motivated by our willingness to provide participants with concrete information about prospective technologies. In their review of creative KTE approaches such as storytelling, the arts or immersive learning, Davies and Powell argue that the use of fiction permits the “exploration of difficult issues in a non-threatening form” and helps researchers better draw in experience and emotion [17: 6]. Along those lines, Cox and colleagues [2] and Kontos and colleagues [19, 28] have conducted ground-breaking research using theatre as a KTE strategy.

**Table 2:** An overview of the three fictional technologies

Enhancement technologies in teenagers—PBF shirt
• A shirt with embedded sensors that provide real-time feedback about the mental state and cognitive performance of the person wearing it
• Used with meditation techniques, the shirt can help one learn about oneself
Preventive interventions for genetically “at risk” adults—Cardiac “rectifier”
• Implantable cardiac “rectifier” that destroys cells genetically susceptible to cause arrhythmia later
• The rectifier transmits data to a centralized system where experts confirm its plan of action
Ageing in a high-tech world—Personal robot
• An assistive personal robot connected to the Internet, which can interact with individuals and the built environment (using face, voice and object recognition)
• The robot is used at home and can “learn” from its owner by asking questions and memorizing responses

The aim of each 3-min video was to describe the fictional technology —providing answers to questions such as “how does it work” and “what does it do”— and to illustrate the prospective context in which it would be used. For each technology, we devised a collective dilemma taking place in 2030 and a personal dilemma arising ten years later. The personal dilemma focused on the specific quests of one main character affected by the fictional technology (e.g., a teenager, a young adult, an elderly person). The collective dilemmas drew participants’ attention to the concrete ways in which society, values and technology influence each other [10, 13]. Each short story depicting a dilemma presented challenges to which participants were likely to relate, both affectively and rationally.

From an evaluative standpoint, the above components are grounded in our hypothesis that a multimedia-based, prospective deliberative intervention can enable individuals to envision and relate to fictional futures, thereby fostering their reflexivity about the social and ethical implications of technological innovation in health. Our decision to integrate sequentially a face-to-face workshop and an asynchronous online forum was informed by the literature. According to Black, participants in asynchronous online forums can take the time to respond without interruption, express their ideas or tell their stories more completely than if they were in a face-to-face interaction [22]. Online tools also offer the possibility to reduce geographical, physical or emotional barriers to discussing sensitive health issues [24–27].

#### Process

The ability of these components to fulfill their goals is, nevertheless, intimately linked to the deliberative processes in which they are embedded [29–32]. In the public involvement literature, there is particular emphasis on participants’ assessment of procedural elements such as “the communication of objectives and tasks to be undertaken” by participants, the adequacy of the information and resources provided and the quality of the deliberation [14: 11]. For Khodyakov, Savitsky and Dalal [26], appraising the level of participant engagement also matters because it affects the extent to which participants may learn through the process and through each other’s contributions. This is particularly salient in online deliberative environments, and it is one of the reasons why our framework takes into account how participants appraise the thoughtfulness of the contributions they have brought to the deliberations, as well as the quality of other participants’ contributions.

#### Expected effects

The outcome criteria used in public involvement evaluation studies tend to focus on measuring the effects of public engagement on participants [14] and this is the focus of the current paper. For the PiiAF Study Group [20], effects may be classified as short- or long-term, positive or negative, intended or unintended. The review conducted by Abelson and colleagues suggests that evaluators of public involvement initiatives have so far favored short-term, positive and intended effects by examining variables such as change in “participants’ views, priorities or values,” “learning about the issue under deliberation” and “competence for future public engagement activity” [14: 11]. Given our focus on health technology design, we expected the components and processes of our intervention to push participants to engage in reflective and critical thinking —pondering what factors make new technologies desirable or undesirable— and learn about their impact on society. As clarified below, our survey was designed to capture such short-term effects, but also offered space for participants to comment on negative or unintended effects.

## Methods

### Study design

**Fig. 2:**
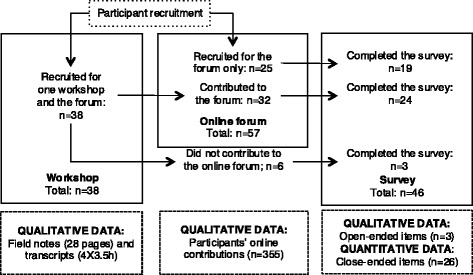
A flow diagram of the participants recruited and of the data gathered

The analyses conducted for this paper follow a mixed-method strategy, defined as a “convergent” study design when “the researcher collects and analyzes both quantitative and qualitative data during the same phase of the research process and then merges the two sets of results” to generate an overall interpretation [33: 77]. The key purpose is to “develop a more complete understanding of a phenomenon” while building on the respective strengths of each method [33: 77]. Figure 2 provides a diagram of the quantitative and qualitative data we gathered. The participant survey contained closed- and open-ended items, which respectively generated quantitative and qualitative data. Additional qualitative data included field notes gathered through non-participant observation of the workshops, transcripts of the workshops and participants’ contributions to the online forum (i.e., their written comments). The Health Research Ethics Committee of the University of Montreal approved the study, all participants provided informed consent and pseudonyms were attributed to all at the beginning of the study.

### Participant recruitment strategies

Multiple recruitment tools and strategies were deployed in parallel to constitute a purposeful study sample [34]. The goal was to reach young adults, adults and people over 60 years old who might share an interest in our three thematic areas, but from across a large range of perspectives and reasoning processes [35, 36]. We reached out to groups that organize reading clubs, conferences, cultural events or training activities for young entrepreneurs, occupational-based networking or retired people. To ascertain the interest and relevance of each organization serving as an intermediary, the recruiter (who also acted as the workshop/forum moderator) contacted each organization by phone or through in person meetings. We circulated an electronic invitation letter through their newsletters and websites as well as through social media. The letter provided links to our study website and information regarding the Health Research Ethics Board approval, and invited potentially interested individuals to contact our recruiter, who then gathered demographic and socioeconomic information about each interested participant through a brief phone conversation. From the pool of interested participants, four groups were assembled using a reasoned sampling technique organized around age, occupational profiles and hobbies [34]. Those who were not available at the day and time set for the workshop were invited to participate in the online forum.

### Structure of the deliberations in the two environments

A professional moderator, with training and experience in group communication, was hired to facilitate all four deliberative workshops, which each lasted for 3.5 h (including a 15-min break) [37, 38]. Once each participant had introduced her- or himself, the first video was shown and then each participant was asked to share with the group 2–3 features of the technology that he/she saw as desirable as well as 2–3 undesirable features. A group discussion ensued focusing on potential ways to improve the technology. The same structure was applied to the other two technologies.

The online forum was hosted on a login/password-secured blog platform (WordPress®) and facilitated by the same moderator. The forum ran over a five-week period, starting after the last workshop. Participants were invited to view a brief animation explaining the study, to read the six scenarios, to view the videos and to respond to questions to kick-start online deliberations. Participants were able to return to the forum whenever they wished, comment on each other’s comments and “like” comments.

### Survey development and administration

The survey was informed by the literature review that we had performed when developing our research proposal for peer-review at the Canadian Institutes of Health Research (CIHR), as well as by assessment frameworks that were published after we received funding [39]. The face validity of our survey was iteratively consolidated. Three members of our research team and two research technicians with expertise in online surveys developed successive versions of the survey. The survey was pre-tested by a graduate student and a postdoctoral fellow who were familiar with the videos and online scenarios. The final validation of the survey covered all of its user- and data-related functionalities, i.e., from filling up the items on a password/login secured website to downloading the whole dataset and transferring it into an electronic database (Statistical Package for the Social Sciences). All open-ended items of the survey involved typing one’s comments into a free text box whereas the close-ended items relied on a 5-level Likert-like scale (an English version of the survey is available here [13]). Specific items were presented to participants depending on the deliberative environment to which they had contributed (workshop and/or online forum). All participants were asked to complete the survey at the end of the forum. Up to three reminders were done by e-mail or phone.

### Field notes, transcripts of the workshops and participants’ online contributions

To examine the context in which the deliberations unfolded, a researcher trained in qualitative research directly observed the workshops. Detailed field notes were recorded on a pre-structured form to describe the characteristics of the interactions between participants (e.g., key contributions, climate, turn taking, flow/intensity of interactions) [38]. The audio recording of each workshop was transcribed verbatim and participants’ contributions to the online forum (*n* = 355) were downloaded from the blog platform into an Excel spreadsheet.

### Data analysis

Our data analysis strategy was structured around our intervention theory (Fig. 1), with the aim of 1) reporting central tendencies in participants’ responses to the survey and 2) fleshing out these findings through the qualitative data [40, 41]. For the survey, descriptive statistics were performed because statistical inference beyond our sample was not justified. We calculated the absolute frequencies and proportions by aggregating four of the five levels of our scale: “totally agree” and “agree” were merged into “agree,” and “disagree” and “totally disagree” were merged into “disagree.” The mid-point of the scale was “more or less agree.” When the possibility to answer “don’t know/doesn’t apply” was provided, we present responses into a DNA category.

Once the quantitative survey findings were complied, we analyzed the qualitative data set to illustrate and complement these findings [33]. Three open-ended items of the survey were directly related to close-ended items and the participants’ free text responses were categorized [42]. The field notes contained a detailed record of how deliberations unfolded in each workshop and important cues regarding each participant’s contribution to the group process and responsiveness to the views shared by other participants [38]. The transcripts of the workshops and the participants’ online contributions to the forum were read carefully several times with the aim of identifying excerpts that illuminated the deliberative processes (excerpts were translated from French to English). The qualitative data were analyzed for their complementarity in the “elaboration, enhancement, illustration, and clarification” of the survey quantitative findings [40].

## Results

### Characteristics of the participants

A total of 38 participants were recruited for the workshops and 32 contributed to the online forum (see Fig. 2). Twenty-five additional participants were recruited for the online forum, for a total of 57 participants. Forty-six participants completed the survey, for a response rate of 73 % (46/63). Twenty-four surveys were completed by participants who contributed to both deliberative environments, 19 by respondents who participated only in the online forum and 3 by participants who attended only a workshop. The frequencies presented below are based on the entire set of respondents (*n* = 46) unless specified otherwise in the text or Tables.

Table 3 summarizes the characteristics of the survey respondents. Among these respondents, 20 % were aged between 18 and 29, 13 % between 30 and 39, 7 % between 40 and 49, 15 % between 50 and 59, 37 % between 60 and 69, and 8 % over 70. More than two-thirds (72 %) were women and for 80 % the highest level of education completed was a university diploma. Levels of income varied with 28 % of respondents declaring a household income below $39,999, 37 % between $40,000 and $59,999 and 35 % above $60,000 (Canadian dollars). 2 Participants’ self-reported level of ease with technology was as follows: 22 % felt more or less comfortable, 59 % mostly comfortable and 19 % very comfortable.

**Table 3:** Characteristics of the participants (*n* = 46)

		Number	Percent
Age	18–29	9	20 %
	30–39	6	13 %
	40–49	3	7 %
	50–59	7	15 %
	60–69	17	37 %
	>70	4	8 %
Gender	Female	33	72 %
	Male	13	28 %
Education	High school	4	9 %
	Collegial	5	11 %
	University	37	80 %
Household income	< $20,000	4	9 %
	$20,000 to $39,999	9	19 %
	$40,000 to $59,999	17	37 %
	> $60,000	16	35 %
Ease with technology	More or less comfortable	10	22 %
	Mostly comfortable	27	59 %
	Very comfortable	9	19 %

### Appraisal of the components: videos and scenarios

Table 4 shows the participants’ appraisal of the multimedia components of our intervention. The vast majority (96 %) considered that the videos helped them understand the fictional technologies and 91 % thought these videos helped them understand the online scenarios. These scenarios helped nearly all participants (98 %) to reflect on the issues raised by the technologies. The online scenarios stimulated discussions for 86 % and 74 % felt concerned by the dilemmas faced by the characters.

**Table 4:** Appraisal of the videos and scenarios

	Total	Agree		More or less agree		Disagree		DNA	
	*n*	*n*	%	*n*	%	*n*	%	*n*	%
The videos have helped me understand the technologies	46	44	96 %	1	2 %	0	0 %	1	2 %
The videos have helped me understand the online scenarios	43	39	91 %	3	7 %	0	0 %	1	2 %
The online scenarios helped me reflect about issues raised by the technologies	43	42	98 %	1	2 %	0	0 %	0	0 %
The online scenarios stimulated discussion	43	37	86 %	6	14 %	0	0 %	0	0 %
I felt concerned by the dilemmas faced by the characters	43	32	74 %	9	21 %	2	5 %	0	0 %

A survey open-ended question offered space for participants to share their comments about the videos. A total of 29 free text responses were categorized as follows: strengths (*n* = 19); strengths and weaknesses (*n* = 4); and weaknesses (*n* = 5). Beyond conciseness, liveliness and clarity, positive comments underscored how effective the videos were for helping non-expert participants to understand how and in what context the fictional technologies would be used. For instance, for one participant videos are:

an effective means to inform about technology and bring many details. It’s very dynamic, characters are brought to life, it’s immediately interesting, and it motivates one to continue the exercise.

Among the weaknesses, participants underlined the loose connections between the videos —explaining how the technologies work— and the social and ethical dilemmas depicted in the online scenarios. Weaknesses also referred to specific aspects affecting plausibility, e.g., videos could have been more futuristic, language used was more formal than is typical in day-to-day conversations, the functioning of one fictional technology was harder to grasp and more details could have been included. Along those lines, one participant commented on the respective effectiveness of the videos in terms of plausibility and levels of detail:

The character of the 1^st^ video was less credible. It’s difficult to believe that Catherine would need a personal robot [note: she appeared healthy]. The 3^rd^ video is the most interesting in my view because the probability that this technology [cardiac “rectifier”] will be developed in the next years is very high. But the problem of arrhythmia should have been better explained at the outset so participants could establish a clearer link with the cardiac rectifier.

### Appraisal of the processes: quality of the deliberations, personal engagement and differences between the face-to-face and online environments

Table 5 provides information regarding the quality of the deliberative processes and how participants assessed their own engagement in these deliberations. Up to 86 % of the respondents felt the moderator contributed to stimulate the group’s reflections and 94 % considered the moderator respected the participants’ opinions. All participants (100 %) considered that they had the opportunity to express themselves freely. The arguments of the other participants appeared well thought out for 81 % of the respondents and group exchanges were felt to have furthered the reflections of 70 %. In terms of personal engagement, 83 % of the participants believed they shared arguments that were well thought out, 89 % were attentive to the views of other participants and 84 % remained interested in the process throughout the study.

**Table 5:** Appraisal of the quality of the deliberations and of one’s engagement throughout the process

	Total	Agree		More or less agree		Disagree		DNA	
	*n*	*n*	%	*n*	%	*n*	%	*n*	%
Moderator									
The moderator contributed to stimulate the group’s reflections	46	39	86 %	3	6 %	1	2 %	3	6 %
The moderator respected the opinions of participants	46	43	94 %	0	0 %	0	0 %	3	6 %
Quality of the deliberative processes									
I have had the opportunity to express myself freely	46	46	100 %	0	0 %	0	0 %	0	0 %
The arguments of the other participants appeared well thought out	46	37	81 %	6	13 %	1	2 %	2	4 %
Group exchanges have furthered my reflections	46	32	70 %	9	20 %	3	6 %	2	4 %
Personal engagement									
My arguments were well thought out	46	38	83 %	8	17 %	0	0 %	0	0 %
I was attentive to the views of other participants	46	41	89 %	3	7 %	2	4 %	0	0 %
I remained interested throughout the experience	43	36	84 %	5	12 %	2	4 %	0	0 %

**Fig. 3:**
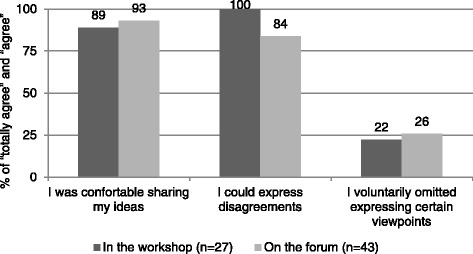
Sharing one’s thoughts in the face-to-face workshop and on the online forum

Six survey items were meant to characterize how comfortable participants were with the sharing of their thoughts in the workshop and online forum. Figure 3 shows the proportion of respondents who totally agreed and agreed with these items. Responses concerning the workshop are those of 27 respondents (24 participated in both study components and 3 only in the workshop) and responses concerning the forum are those of 43 respondents (24 participated in both study components and 19 only in the online forum). The level of ease was similar in the two deliberative environments: participants were comfortable sharing their ideas (respectively 89 % in the workshop and 93 % in the forum), felt they could express disagreements (100 %; 84 %) and close to a quarter (22 %; 26 %) voluntarily omitted expressing certain viewpoints.

To provide nuance to the way disagreements were shared in each deliberative environment, we draw from our field notes, the transcripts from the workshops and the participants’ online contributions. According to our field notes, participants were motivated to attend the workshop because they were intrigued by how technologies might shape the future. In each workshop, participants were attentive, respectful of each other and disciplined. The moderator, a group communication expert, had created a preliminary contact by phone with each participant and, as the workshop progressed, a form of convivial authority over the group was palpable. Our team explicitly hired a senior moderator who possessed the skills required to create a group climate where participants would feel safe sharing their thoughts. The excerpt from the transcript below shows how short probes by the moderator and respectful turn taking among participants created a legitimate space for disagreements:

MAUDE (*pseudonyms are used throughout the text*): I should share a reflection as a user, this is the task you gave us, hum? Well, I buy it right now! [laughs].

MODERATOR: Ah, OK… you want a crate [laughs].

MAUDE: Ah, this is fantastic. Yes, absolutely. Especially since it’s inserted through the large vessels, it even travels by itself … [laughs].

MODERATOR: And, by and large, what is it that makes you … praise it so much? Without getting into too much detail …

MAUDE: Well, because it’s… it intervenes before the problem. It detects before the means we currently know can do it and this is what makes it fantastic. We know that cardiac problems, at the moment they have been detected, have already done some damage, most of the time. [With this technology], it’s well before.

MODERATOR: OK. So it appeals to you.

MAUDE: Two crates, please! [laughs]

MODERATOR: OK. Perfect. Thanks. We continue with Florence.

FLORENCE: There’s a problem with the fact that, at the outset, they say to the client: “According to our genetics analysis, you’re at risk of a cardiac problem within 15-20 years.” I find that … by using this gadget that will destroy certain cells that are —if I understood well— potentially sick. It’s like nothing has been declared yet. And they will … they’ll do the treatment during 15–20 years without being certain … ishh… I find it very … I struggle with this. Myself, no…

MODERATOR: Serious doubts?

FLORENCE: It’s an arrhythmia, it’s not … clear death within 15–20 years. It’s… there should have … for me to buy into this, there must have been a more terrible, certain diagnostic. So, no, I don’t think I’d do this. I don’t buy two crates! [laughs].

The online forum also enabled participants to explicitly agree or disagree with each other but we did not observe any “heated” exchanges. The two contributions below are illustrative of the way participants interacted online, where the second participant used a polite “I agree, but…” response in order to share a complementary viewpoint:

JOSEPH: I feel an enormous resistance to the proposed scenario. […] It sounds like a toy coming from inventors in need of gadgets. […] I can’t prefer the robot over an accompanying person, a human contact, a person who drives a car, who accompany me for doing the groceries, and prolong transient elements of an active life … […] I’d rather propose a technical collegial-level training for geriatric caretakers (Forum Group, 280).

FABIEN: I very much agree with your comments. However, if a machine could discharge humans from performing daily tasks that are constraining and tedious, we could liberate more time for human exchanges (F28 Group, 347).

**Fig. 4:**
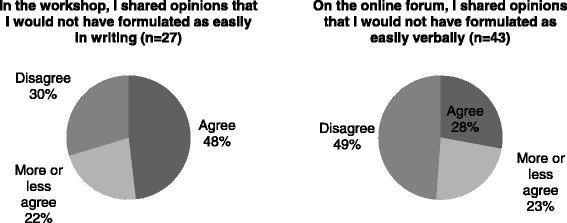
Ease with the verbal and written formats of the deliberations

The moderator made brief online interventions every other day, inviting participants to elaborate on their views. Yet, one aspect that did not work as well as we had expected is that online interactions among participants remained moderate: about a third of the participants (32 %; 18/57) replied to another participant’s contribution and half (51 %; 29/57) “liked” another participant’s contribution. In fact, an important distinction between the two deliberative environments was the format in which participants were asked to share their thoughts: verbally or through written comments. Two survey items sought to measure the level of ease with these formats. Figure 4 shows that 48 % (13/27) of the respondents who attended the workshops believed they shared opinions that they would not have formulated as easily in writing and less than a third (30 %; 8/27) disagreed with this statement. Up to 28 % (12/43) of those who contributed to the online forum shared opinions they would not have formulated as easily verbally and close to half (49 %; 21/43) disagreed with this statement. These data suggest that the written format had an added value for 28 % of the participants.

Two survey items explored further the distinctions between the two environments. To the close-ended question “did you prefer one of the two deliberative environments?”, 88 % (21/24) of those who participated in both components indicated the face-to-face workshop, 4 % (1/24) the online forum and 8 % (2/24) had no preference. Participants could then explain the rationale underlying their preferences in a free text box. As participants did not mention *weaknesses* related to the workshop, we categorized 35 responses as follows: strengths of the workshop (*n* = 22); weaknesses of the forum (*n* = 9); and strengths of the forum (*n* = 4).

The *strengths* associated with the workshop underscored that participants not only enjoyed exchanging ideas with other persons face-to-face, but they also felt that a format where one is alternatively listening and talking and where a moderator intervenes in the group process was more conducive to eliciting one’s thoughts. This synchronous group dynamic was well summarized by one respondent:

The workshop is more dynamic. It’s easier to react on the spot. The contribution of the moderator is to help participants clarify the opinions being shared when needed. We get the overall picture of the opinions and our own opinion evolves, this is in contrast to the forum where one must read everything, which takes much more time.

The *weaknesses* related to the online forum indicated that it was a cognitively more demanding deliberative environment, considering the two tasks of contributing and reading. One respondent underscored that “it’s easier to tell one’s thoughts than to write them down.” For another, the “comments evolved quickly” and this increased the difficulty of “knowing which comments to focus on.” The reader’s difficulty was increased by online contributions that suffered from grammatical and orthographic errors 3: “reading the opinions of certain participants was at time more arduous (thoughts are not well structured, therefore difficult to follow),” hence “we lose less time when we listen during a workshop than when we read in an online forum.”

The *strengths* of the online forum highlighted the contributor’s standpoint; it provided more time to reflect about the dilemmas before responding, it enabled participants living outside the city to contribute and those who were familiar with electronic communication knew how to summarize their thoughts in fewer words.

Finally, one respondent eloquently described why using both kinds of environments may confer more depth to one’s thinking: “The two environments enable different forms of interaction, one that is more dynamic and the other that is more reflective. This combination enables to make deeper reflections and think about their emotional impact.”

### Perceived effects

Table 6 shows participants’ appraisal of the extent to which they engaged in critical and reflective thinking and learned about technological change in health. Up to 85 % of respondents reflected more about the pros and cons of technologies, 85 % discovered effects of the technology that they had never before imagined and only 30 % have looked for additional information on the topics discussed. In terms of learning, nearly all participants reported knowing more about the way technologies may transform society (94 %) and 85 % knowing more about the way values may influence technology design and use.

**Table 6:** Perceived effects of the deliberative intervention

	Total	Agree		More or less agree		Disagree		DNA	
	*n*	*n*	%	*n*	%	*n*	%	*n*	%
Critical and reflective thinking									
I reflected more about the pros and cons of technologies	46	39	85 %	5	11 %	1	2 %	1	2 %
I discovered effects of technology that I had never imagined	46	39	85 %	5	11 %	2	4 %	0	0 %
I looked for additional information on the topics discussed	43	13	30 %	13	30 %	13	30 %	4	10 %
Learning									
I know more about the way technologies may transform society	46	43	94 %	2	4 %	1	2 %	0	0 %
I know more about the way values may influence technology design and use	46	39	85 %	4	9 %	2	4 %	1	2 %

At the end of the survey, respondents were asked whether they had something to add about their experience with the study. We categorized 34 participants’ free text responses as follows: reflections (*n* = 11); learning (*n* = 6); challenges (*n* = 8); and enthusiasm (*n* = 8). Participants shared *reflections* that addressed the impact of technological change on society, the hard collective choices that need to be made, the tension between individual autonomy and public policies and the tension between privacy and scientific advances. Comments that summarized participants’ *learning* stressed the importance of bioethics, principles regarding how technology should be designed, used and assessed, and the need for user support and training. The *challenges* included the difficulty of projecting oneself into the future, the presumptions underlying our scenarios and the time and efforts required to comment on six complex dilemmas and to read participants’ online contributions. Participants conveyed their *enthusiasm* by stressing they would repeat the experience, the richness of the contributions that were shared and the importance of public engagement initiatives like ours, which was considered an “eye-opening” deliberative experience.

Before closing the online forum, we created a page where participants could share a “final word.” The comment bellow illustrates the collective context in which our study took place:

Good evening to Jean [the moderator], to his team and to all forum participants,

I really loved my experience as much in the group as on the blog. […] It wasn’t always easy to respond to the futuristic hypotheses and make good reflections out of them, but the experience has enabled me to put my neurons to work without barriers and to go beyond preconceived ideas about the future and new technologies. This exercise has enabled us to think about the future and try to imagine it freely without consequences […] (Laura, F8 Group).

The notion that our intervention brought participants to engage in deliberations “without consequences” highlights its prospective nature as well as the earnest playfulness that characterized the deliberations.

## Discussion

This rigorous, small-scale study brings a three-fold contribution to the growing body of methodological literature that examines how informed deliberations among non-experts can be better supported (see Table 7). Along those lines, we clarify below key insights from our study, offering guidance for further research.

**Table 7:** What this study adds to current knowledge

• Making explicit one’s intervention theory helps to fill a research gap by producing knowledge on how the components and processes of tailor-made interventions are linked to their outcomes
• This study confirms the methodological feasibility of a deliberative intervention that relies on videos and scenarios to enable productive deliberations among non-experts
• Our findings help understand why face-to-face and online deliberations need to be combined if the goal is to increase deliberative depth and foster learning across groups

### Linking components, processes and outcomes through the intervention theory

As recommended by the PiiAF Study Group, we structured our mixed-method evaluation by making our deliberative intervention theory explicit [20]. This framework enabled us to organize different data sources in order to illuminate the expected linkages between the goals of each component of our intervention, its deliberative processes and hoped for outcomes. Such a theoretically-grounded assessment contributes to filling a gap in the literature that seeks to improve the design and assessment of tailor-made public engagement and KTE interventions [14, 17, 19].

Pursuing developmental public engagement objectives, our deliberative intervention was characterized by a reflective playfulness, which was coherent with our intervention theory and certainly in contrast with focused public involvement initiatives that, for instance, ask participants to think as if they were decision-makers (i.e., priority-setting exercises) [7] or reach a verdict (i.e., citizens’ juries) [45]. The survey findings indicate that our deliberative processes were well moderated, adequately structured and productive; the moderator contributed to stimulating the group’s reflections (86 %), participants had the opportunity to express themselves freely (100 %), the arguments shared appeared well thought out (81 %), participants were attentive to each other’s views (89 %) and group exchanges furthered their reflections (70 %). Respondents considered our deliberative intervention to have fostered their critical and reflective thinking and learning (ranging from 85 % to 94 %). They also shared through free text responses concrete examples of reflective thinking and learning. Given the premise of our study, one may have hoped the intervention to trigger the desire to know more about health innovation and thus observe a higher proportion of participants having looked for additional information on the topics discussed (30 %). While participants conveyed their enthusiasm toward our study’s purpose, they also mentioned that the time and effort required to comment on six complex dilemmas was one of its challenges. Overall, our findings indicate that our intervention succeeded in prompting reflective and critical thinking about sociotechnical change in health.

### Videos and scenarios enable productive public deliberations

One key novel contributions of our intervention lies in its use of videos and scenarios that draw on fiction. None of the 34 studies reviewed by Abelson and colleagues in 2011 [14] and of the 62 deliberative events reviewed by Degeling and colleagues in 2015 [18] relied on the use of multimedia material. By showing that our participants found the videos and online scenarios helpful and stimulating (in proportions ranging from 86 % to 98 %), our survey data lend support to the development of tools that seek to reduce the expertise asymmetry characterizing public deliberations around complex health issues [3, 6]. Such tools may help rethink the role experts play in public engagement methods such as citizens’ juries and may offer new avenues for KTE. In a review of 66 articles reporting on KTE impact, the most commonly described applications were “printed materials such as booklets or guideline checklists (reported in 66 % of the articles), and interactive in-person workshops (reported in 50 % of the articles)” [21: 35]. Our study thus contributes to current scholarship by confirming the methodological feasibility and relevance of a deliberative intervention that relies on multimedia-based tools to support informed deliberations among non-experts. Acknowledging that our participants’ free text responses concerning the videos identified more strengths than weaknesses, our findings can inform those who would like to develop similar interventions.

### Face-to-face and online environments support different kinds of deliberation and need to be combined in meaningful ways

The issue of whether online tools can support effective deliberations has attracted the attention of both practitioners and scholars of public involvement [22, 25, 26]. Our findings indicate that our participants were comfortable sharing their ideas in both deliberative environments (89 % vs. 93 %) and could, with some variation between the two environments (100 % vs. 84 %), express disagreements, which is a desirable attribute if one wishes to provide more depth to the deliberations [29, 31, 32]. According to Khodyakov, Savitsky and Dalal [26: 2], online tools can allow participants to judge arguments “based on the soundness of arguments, rather than participants’ personalities” because of their anonymous nature. Around a quarter of our participants declared having voluntarily omitted expressing certain viewpoints in both environments (22 % vs. 26 %). Since there could be legitimate reasons for refraining from sharing certain views as much as unsuspected barriers, the contexts in which anonymous deliberations are considered relevant require further attention.

Carman and colleagues recently conducted an ambitious five-arm randomized controlled trial (RCT) to examine the quality and impact of four deliberative methods against a control group (reading materials) [25]. One of the methods they tested relied on Online Deliberative Polling® (ODP), which consisted in four weekly 1.25 h online synchronous deliberative sessions. These authors compared the ODP method to an in-person method of similar intensity. This comparison did not show a statistically significant effect on the knowledge and attitude outcomes, but showed “dramatic differences” in deliberation quality and experience measures with in-person participants reporting significantly higher scores than ODP participants [25]. The investigators were unable to determine if the characteristic that contributed to a less positive experience was its online format or its “passive facilitation” [25: 109].

Like Carman and colleagues, we recruited participants who were comfortable with online tools and who belonged to different age groups. In contrast to their RCT though, our intervention integrated sequentially two types of deliberative environment and we could explore their respective value from the participants’ standpoint. All but three participants preferred the face-to-face workshop and no weaknesses were mentioned for this type of environment. For participants, debating within a group that is competently moderated is both enjoyable and conducive to eliciting one’s viewpoint. Our findings thus concur with Boyko and colleagues for whom:

a skilled, knowledgeable and neutral facilitator for a deliberative dialogue is necessary to enable structure and process, while encouraging mutual understanding and innovative thinking within the group. Specific skills that a facilitator requires include keeping track of the conversation, pulling together different strands of the conversation and ensuring all participants have the opportunity to contribute [16: 1949].

Although it is possible, in principle, to reproduce high-quality facilitation online, one may wonder whether well-structured face-to-face deliberations would always prove more appealing to participants. The strengths and weaknesses that participants identified for both environments suggest that the contributor’s and reader’s tasks were more demanding in the online forum. In addition, some participants may have felt comfortable with expressing their views through a keyboard even though they did so in a written French that was, at times, considered by other participants to be of uneven quality. Our study thus suggests that online deliberations should be designed and assessed recognizing the two sides of the coin: the contributor’s self-perceived ability to share comments in writing —which may be over- or under-estimated— and the time and efforts required on the part of the reader to decipher these comments. This is an important issue because it is through the interactions it supports between various participants that a public engagement intervention can fulfill its ultimate goal [14, 30]. Although we share the cautionary stance of Carman and colleagues regarding the “quality and experience of online deliberation,” we have reservations about the notion that online methods could be used “in situations where gathering people in an in-person venue is difficult or impractical” [25: 109]. The risk we see is that online tools would be used as a standalone, second-best method, which may increase civic inequalities [27] in countries with a geographically dispersed population. If the goal is to increase deliberative depth and foster a strong engagement by all participants, then online tools need to be embedded within a deliberative intervention that includes face-to-face venues and fosters learning across various groups [26, 27].

### Strengths and limitations

Six workshop participants chose not to pursue the online forum and the reasons were either lack of interest or time, or not having any new ideas to share, but we have no information about participants who did not respond to the survey. We followed a rigorous, iterative process to ensure the face validity of our survey items, which were either based on existing tools or created to capture the specificities of our intervention. Yet, self-reported measures like the ones we used suffer from limitations and, although the response rate to our survey was very good, the descriptive statistics presented in this paper cannot be generalized beyond the group who responded to the survey. We favored purposive over stratified random sampling not only because qualitative research principles predominate in our broader study, but also because random sampling was not applicable to our four workshops. A third of our sample (33 %) included individuals who were below 40 year-old and 45 % were over 60; it was comprised of educated individuals and more women than men agreed to participate. This type of sample is often found in public involvement studies [45].

Because we triangulated different sources of data, the internal validity of our findings is high. We rigorously gathered qualitative data, which enabled us to put empirical “flesh around the bones” of the survey data and more fully address the linkages between the components, processes and perceived effects of our intervention. We thus believe that meaningful comparisons can be made with published studies. Overall, our rigorous, small-scale study provides original findings that lend support to, but also complement current knowledge, thereby offering precious insights for policy, practice and further research [46: 4].

### Further research

We concur with van Eerd and colleagues who stress the need “to continue measurement research and development of KTE evaluation instruments” in order to develop pre/post instruments that can measure meaningful change [21: 80]. In their systematic review of the quality and types of instruments used to assess KTE impact, these authors found that up to 55 % of the 54 retrieved quantitative studies did not report on the measurement properties of the instruments the investigators had created or used for the specific context of their evaluation [21]. It would also be relevant to conduct comparative studies on the respective benefits of different tools (e.g., printed material, online tools, expert testimonies) to support and stimulate deliberations among non-experts.

## Conclusion

While those who design technologies make several social and ethical assumptions on behalf of users and society more broadly [2, 5, 6], there are very few tools to examine how the public define and appraise the desirability of health innovations. By designing and assessing a multimedia-based intervention meant to support prospective deliberations among non-experts, this methodological paper represents a preliminary step toward bridging this gap. Beyond confirming that members of the public are eager to contribute to deliberations around complex health innovation issues, our study showed: 1) the usefulness of making one’s intervention theory explicit in order to assess how the components and processes of the intervention are linked to its outcomes; 2) the feasibility of using videos and online scenarios that draw on fiction to support productive public deliberations; and 3) the need to meaningfully combine face-to-face and online deliberative environments. Notwithstanding the areas for improvements that participants identified, our intervention succeeded in prompting reflective and critical thinking and learning about sociotechnical change in health.

## Abbreviations

DNA: Do not apply
KTE: Knowledge transfer and exchange
ODP: Online deliberative polling®
PiiAF: Public involvement impact assessment framework
RCT: Randomized controlled trial

## Footnotes

1 See [13] for a description of the literature review and foresight policy briefs we used. The draft scenarios were reviewed by an external Expert Committee comprised of members with expertise in: family medicine, engineering, nursing, pediatric psychiatry, bioethics, geriatric care, genetics and public engagement (see Acknowledgments).

2 As a comparator, 29.4% of Quebec residents between 25-64 year-old hold a university diploma and this proportion rises to 46.5% in Montreal, the largest city [43]. With an average revenue of $36,439 per habitant, the province of Quebec ranks 12th in the country, that is, the second lowest [44].

3 This problem may be more acute with languages like French where many rules apply to adjectives and verb tenses, including the need to adequately handle gender and plural forms. The need to translate our excerpts from French to English precludes us from fully illustrating the problem that orthographic and grammatical errors may cause in online forums, but the excerpt below is one example: “I think robots can offer the advantage of being very present, night and day, and to fulfill tasks that elderly persons are no longer able to do or that causes [sic] them pain, but also insure safety of the person and assistance when moving in the bathroom, etc. Nonetheless, it’s very obvious that a robot will never replace entirely a human being and I believe that certain things must be left to humans, like medications administration, care, certain health state assessments, etc. […] I find it really great that the code of the robots be open source, because it will be possible to individualized [sic] them well and to develop for free much material and improve the existing code, which could potentially reduce certain costs for the state [sic] (Carine, M3 Group)”.
